# Selective Growth of WSe_2_ with Graphene Contacts

**DOI:** 10.1186/s11671-020-3261-y

**Published:** 2020-03-12

**Authors:** Yu-Ting Lin, Xin-Quan Zhang, Po-Han Chen, Chong-Chi Chi, Erh-Chen Lin, Jian-Guo Rong, Chuenhou Ouyang, Yung-Fu Chen, Yi-Hsien Lee

**Affiliations:** 1 Department of Physics, National Central University, Zhongli, Taoyuan, 32001 Taiwan; 2 Department of Materials Science and Engineering, National Tsing Hua University, Hsinchu, 30013 Taiwan

**Keywords:** Contacts, WSe_2_, Electronics, Heterostructures, Interfaces

## Abstract

Nanoelectronics of two-dimensional (2D) materials and related applications are hindered with critical contact issues with the semiconducting monolayers. To solve these issues, a fundamental challenge is selective and controllable fabrication of p-type or ambipolar transistors with a low Schottky barrier. Most p-type transistors are demonstrated with tungsten selenides (WSe_2_) but a high growth temperature is required. Here, we utilize seeding promoter and low pressure CVD process to enhance sequential WSe_2_ growth with a reduced growth temperature of 800 °C for reduced compositional fluctuations and high hetero-interface quality. Growth behavior of the sequential WSe_2_ growth at the edge of patterned graphene is discussed. With optimized growth conditions, high-quality interface of the laterally stitched WSe_2_-graphene is achieved and characterized with transmission electron microscopy (TEM). Device fabrication and electronic performances of the laterally stitched WSe_2_-graphene are presented.

## Introduction

Monolayer van der Waals materials, such as graphene and transition metal dichalcogenide (TMD), exhibit excellent electronic performances and atomically thick body without dangling bonds on the surface, which offers potential solutions for fundamental limit of channel materials in Moore’s law, such as short channel effects and various challenges in the scaling [1, 2]. In the past decade, nanoelectronics of two-dimensional (2D) materials and related applications are highly hindered by critical contact issues with the semiconducting TMD monolayers due to significant Fermi level pinning effect from the defects involved in the synthetic, fabrication, and integration processes [3–6]. Considerable efforts, including phase engineering of the channel materials (from semiconducting 1H phase to conductive 1T phase) [7], geometry of contacts [8–11], and interface engineering with graphene buffer layer [12, 13], are carried out for essential electronic performances with improved contact properties.

Recently, integration of conductive graphene and semiconducting TMD for improved contacts and novel properties is realized by direct growth of TMD using chemical vapor deposition at the edge of artificially patterned graphene [14–21]. Heterojunctions among different 2D materials enable essential multifunctionality of the monolayer channels for broader capacity and integration [22–27]. Weak tunneling barrier is achieved at the heterojunction of the laterally stitched MoS_2_-graphene, enabling inverter and negative-AND (NAND) gates for a complete set of logic circuits based on 2D materials [16, 17]. The next essential goal is to realize basic electronic units of complementary metal-oxide semiconductor (CMOS) inverters and other logic circuits with scalable 2D materials. Towards this goal, however, it remains a long-lasting challenge on selective and controllable fabrication of p-type or ambipolar transistors with a low Schottky barrier [28]. Most p-type transistors are demonstrated with tungsten selenides (WSe_2_) but a high temperature is required for the WSe_2_ growth because of a higher evaporation temperature of the WO_3_ precursor [29–31]. A low temperature synthesis of the sequential monolayer growth at the pre-patterned 2D materials is mainly achieved with Mo-based TMD.

Here, we utilize seeding promoter and low pressure CVD process to enhance sequential WSe_2_ growth with a reduced growth temperature for reduced compositional fluctuations and high hetero-interface quality [32, 33]. Growth behavior of the sequential WSe_2_ growth at the edge of patterned graphene is discussed. With optimized growth conditions, high-quality interface of the laterally stitched WSe_2_-graphene is achieved and studied with TEM. Device fabrication and electronic performances of the laterally stitched WSe_2_-graphene are presented.

## Method/Experimental

### Synthesis of WSe_2_ and Graphene

Large-area WSe_2_ films were synthesized on sapphire and SiO_2_/Si substrates in the furnace. Before growth process, the substrates were cleaned with acetone, isopropanol, and then water for 10 min, respectively. Perylene-3,4,9,10-tetracarboxylic acid tetrapotassium salt (PTAS) was uniformly coated on the substrate surface as seeding promoters to enhance the activity and growth rate of the monolayers. High purity solid precursors of WO_3_ (Alfa Aesar, 99.9995% CAS#1313-27-5) and Se (Sigma-Aldrich, 99.5% CAS#7704-34-9) were placed in two ceramic crucibles, and the substrates were placed face up and next to the WO_3_ powder. The WSe_2_ samples were synthesized during 800~900 °C for 10 min with a heating rate of 30 °C min^−1^ and under a mixture of N_2_/H_2_ flow at 1.2 Torr. Graphene is synthesized on Cu foil at 1000 °C for 10 min with a heating rate of 30 °C min^−1^ and under a mixture of CH_4_/H_2_ flow at 4 Torr. The pattern graphene is carried out by e-beam lithography and oxygen plasma etching.

### Device Fabrication

The graphene-WSe_2_ devices were fabricated without sample transfer. E-beam lithography process was performed to define the electrodes on the patterned graphene layer. A thin metal layer of Pd (40 nm) was deposited using e-beam evaporation and a following lift-off process was carried out in acetone. Encapsulation layer and gate dielectric of the device are fabricated by using atomic layer deposition (ALD) of thin Al_2_O_3_ films (50 nm). A thin metal of Pd (40 nm) was deposited on the dielectric layer to use as the gate electrodes. To improve electronic performances, the devices are annealed at ~ 120 °C for ~ 12 h in a vacuum environment of ~ 10^−5^ Torr.

### Characterizations

Raman spectra and photoluminescence (PL) were obtained by commercial confocal Raman spectroscopy (Micro Raman/PL/TR-PL Spectrometer, Ramaker, Protrustech). Wavelength and spot size of the laser are 532 nm and 1–2 μm, respectively. Typical gratings were used with 300 g/mm for PL (low resolution) to get broadband spectrum and (high resolution) 1800 g/mm for Raman signals to get detail information of material. The TEM samples were prepared by using standard PMMA transfer technique to place the graphene-WSe_2_ nanosheets onto the holey-carbon Cu grid. The TEM images were performed at an accelerate voltage of 80 kV (Cs-corrected STEM, JEOL, JEM-ARM200F). The electrical measurements were measured using an Agilent B1500a Semiconductor Device Analyzer.

## Results and Discussion

**Fig. 1:**
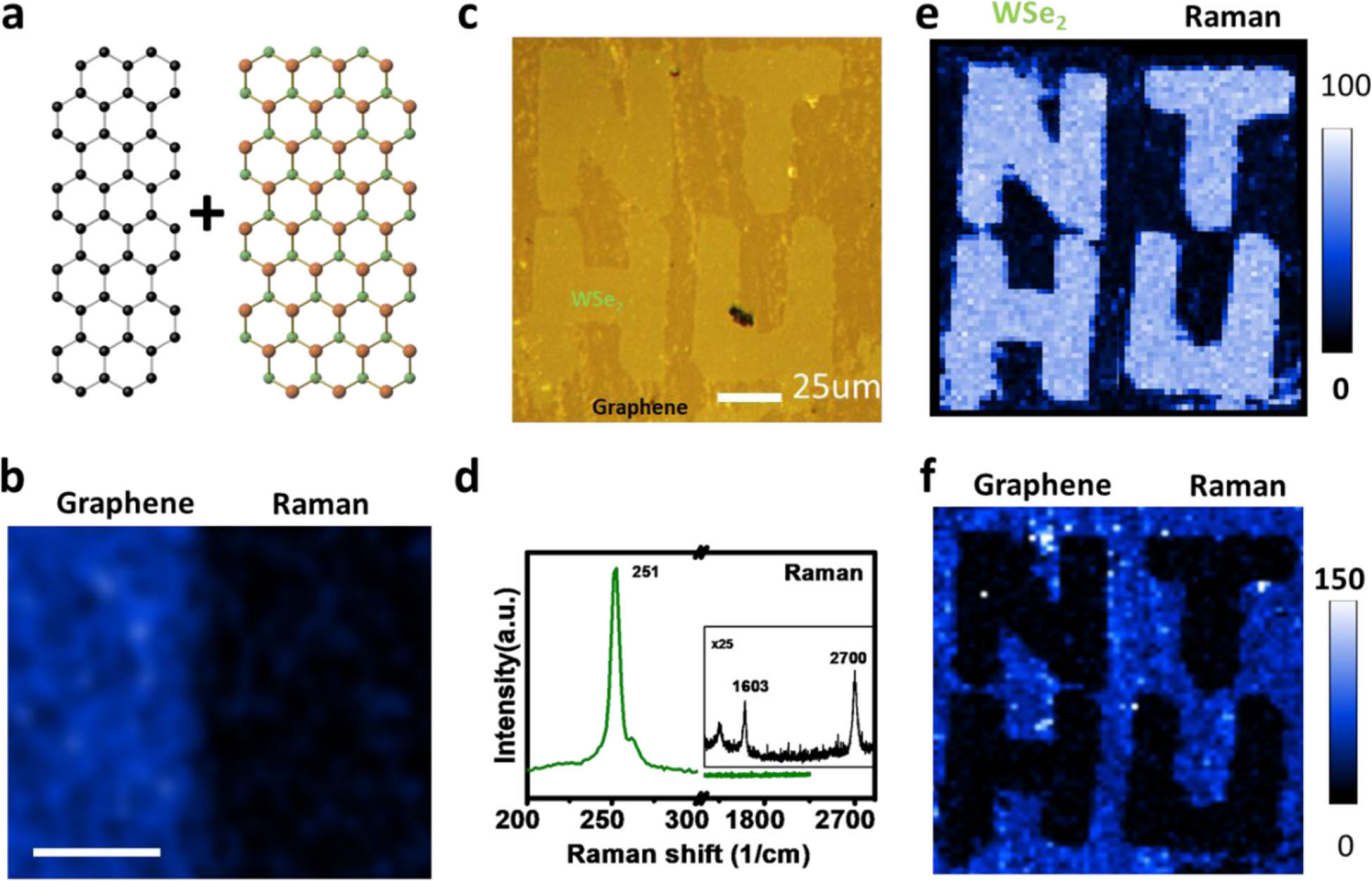
Controlled growth of the WSe_2_ at patterned graphene. **a** Schematic of the laterally stitched WSe_2_-graphene synthesis. **b** Raman mapping for the G’ band of the graphene and **c** AFM image of the patterned growth of the WSe_2_-graphene. **d** Raman spectra of the E_2g_ mode (WSe_2_—blue) and the G’ band (graphene—green) in **c**. Raman mapping of **e** the E_2g_ mode of the WSe_2_ and **f** the G’ band of the graphene in the monolayer heterojunction

To control the synthesis of the lateral heterojunction of graphene and WSe_2_, sequential growth of the monolayer TMD at the graphene edges is demonstrated in Fig. 1a. Monolayer graphene is first grown on a copper foil and later transferred onto a fresh sapphire substrate by using standard PMMA-assisted transfer method. Conventional e-beam lithography and O_2_ plasma etching processes are conducted to define the region for sequential growth of the monolayer WSe_2_. Direct synthesis of monolayer WSe_2_ at the edges of patterned graphene on sapphire substrate is achieved by low pressure CVD with PTAS as seeding promoters. More detailed information on the synthesis is described in the “Method/Experimental” section. In Fig. 1b, Raman mapping of the G’ band in the laterally stitched graphene-WSe_2_ displays a uniform contrast, which confirms a reduced damage of the pre-patterned graphene after the sequential CVD synthesis of the WSe_2_ growth. In Fig. 1c, AFM image of the patterned growth of the graphene-WSe_2_ indicates a smooth surface morphology of the monolayer heterojunction. Figure 1d presents the Raman spectra of the E_2g_ mode (WSe_2_—blue) and the G’ band (graphene—green) as the labels in Fig. 1c, which are consistent with the reported studies [34]. To illustrate uniformity of the as-grown heterojunction, Raman mapping of the patterned graphene-WSe_2_ is shown in Fig. 1 e and f, respectively. A uniform contrast of the Raman intensity in the mapping images is clearly observed, suggesting controllable synthesis on heterogeneous growth of high-quality monolayer WSe_2_ at the edges of the pre-patterned graphene.

**Fig. 2:**
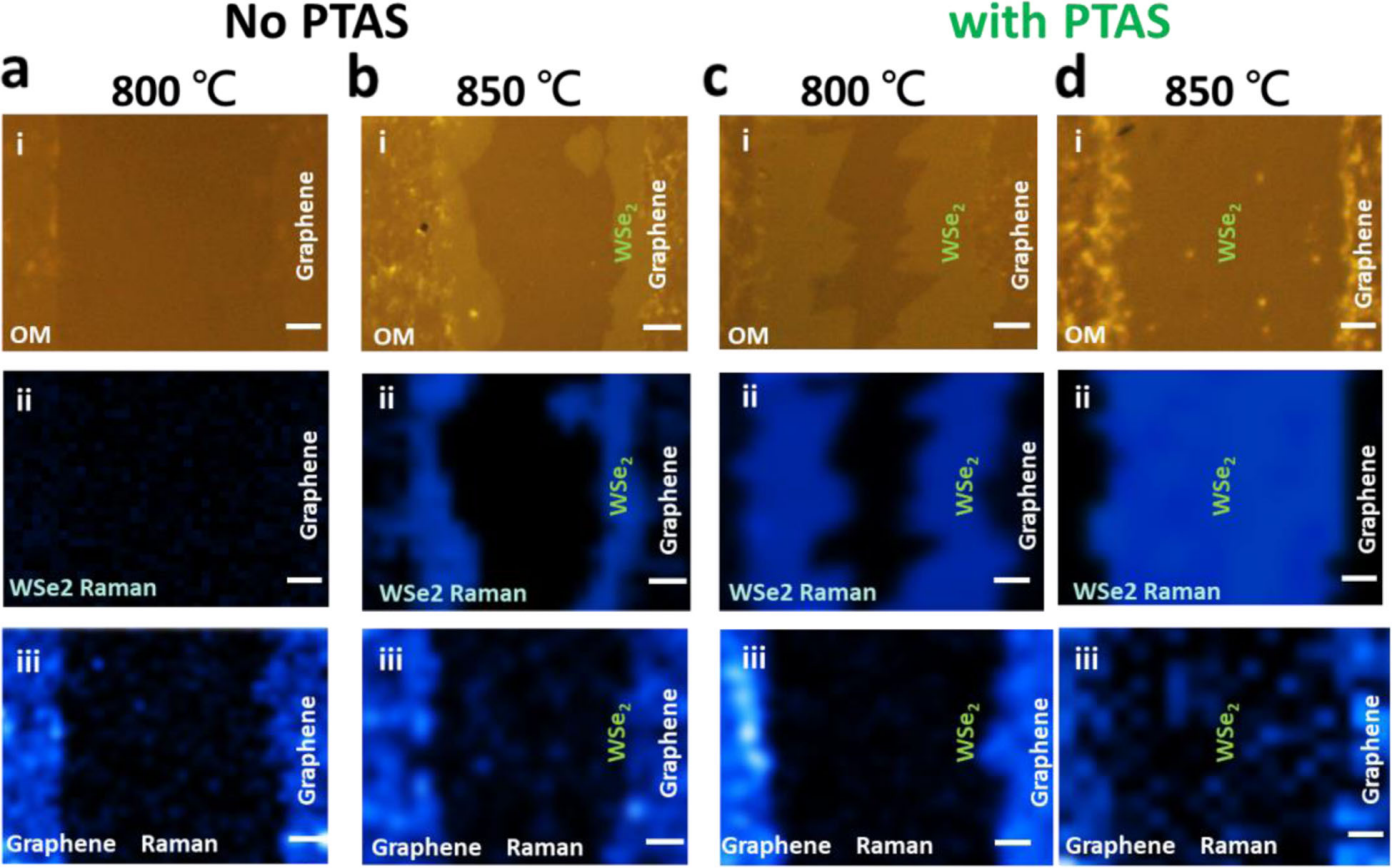
Temperature-dependent WSe_2_ growth with seeding promoter: Optical images, Raman mapping images of the A_1g_ mode (WSe_2_) and the G’ band (graphene) of the samples that are synthesized at different temperatures **a**, **b** without and **c**, **d** with PTAS as seeding promoter

To clarify growth behavior of the stitched graphene-TMD, the WSe_2_ synthesis at the patterned graphene is carried out with and without promoters. Figure 2 a and b suggest the WSe_2_ growth at different temperatures without PTAS as seeding promoter. Above 850 °C, the sequential growth of the WSe_2_ appears at the graphene edges. A high growth temperature for WSe_2_ growth is required due to reduced gaseous reactants for the solid precursor of the WO_3_, as elaborated in previous papers [29–31]. A macroscopically smooth boundary of the as-grown WSe_2_ implies random distributed and small size of grains. In contrast, the sequential WSe_2_ growth at different temperatures with PTAS as seeding promoter is presented in Fig. 2 c and d. The PTAS promoters significantly reduce growth temperature for perfect sequential WSe_2_ growth at the graphene edges with larger domain sizes, which is similar to the growth behavior in the TMD-TMD heterojunctions [22]. After the sequential WSe_2_ growth at 800 °C, observation of a uniform contrast and higher intensity in Raman mapping of the G’ band (graphene) indicates a reduced damage of the graphene because of the low temperature growth. With increased temperature, a continuous WSe_2_ film fills in the patterned regions with ideal contact to the edges of the patterned graphene (Fig. 2d). Note that a larger domain size with a clear triangular shape of the monolayer WSe_2_ stitched to the edges of the graphene (Fig. 2c), suggesting a better quality of the sequential WSe_2_ growth. With optimized growth conditions on seeding promoters and temperature, scalable and high-quality monolayer WSe_2_ is realized by the LPCVD system as presented in the supporting information (Additional file 1: Figure S1). It is noteworthy that the sequential TMD synthesis at the edges of patterned graphene is universally observed in other heterojunctions of different TMD and graphene as shown in the supporting information (Additional file 1: Figure S2).

**Fig. 3:**
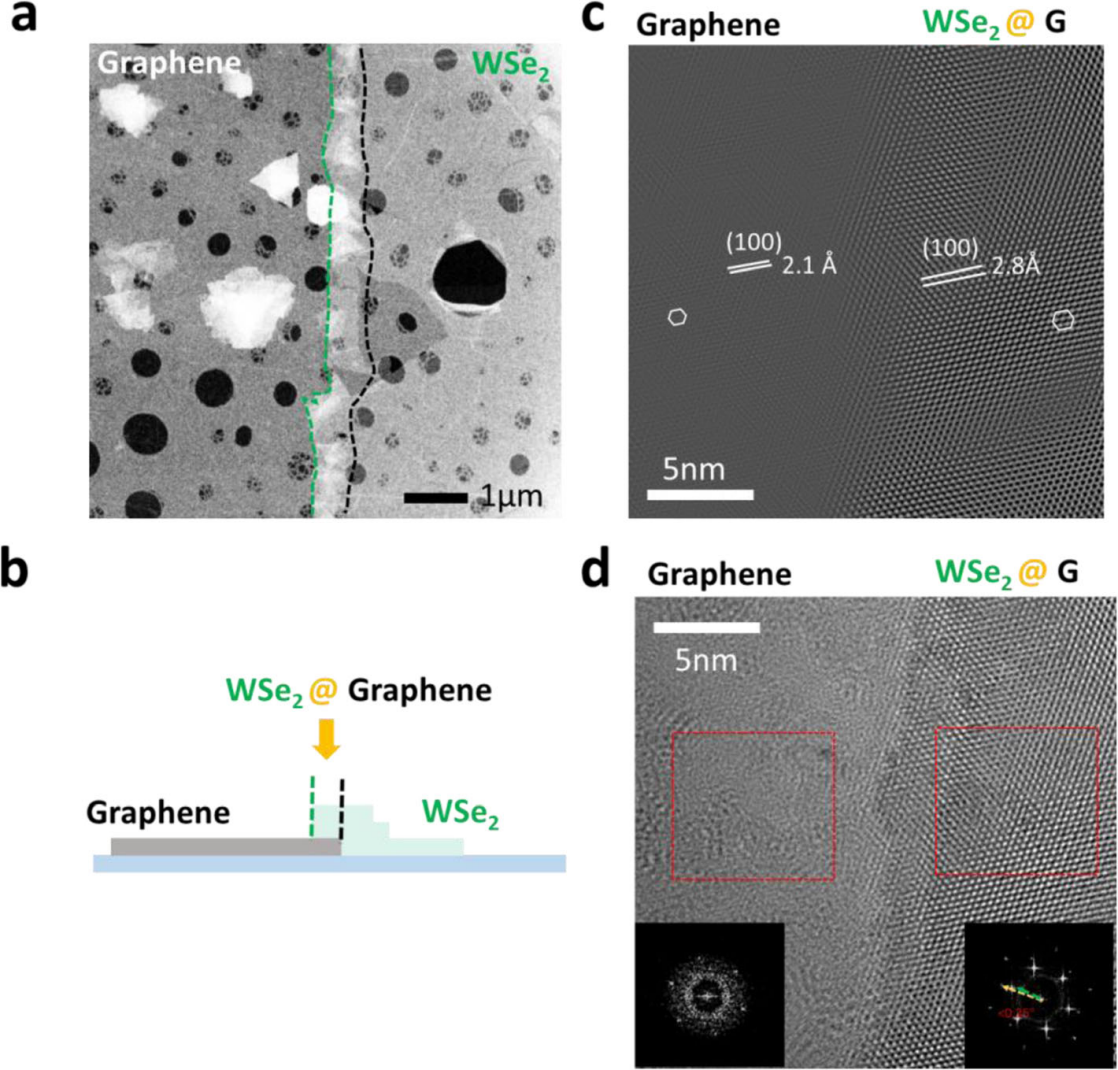
TEM characterization of heterojunction of the laterally stitched graphene-WSe_2_. **a** Low magnification image, **b** schematic illustrations, **c** simulated, and **d** observed HRTEM images of the heterojunction of the graphene-WSe_2_. The right inset shows FFT image of the overlap region of the stacked WSe_2_ on graphene, while the left inset displays that of the graphene. Raman mapping of **e** the E_2g_ mode of the WSe_2_ and **f** the G’ band of the graphene in the monolayer heterojunction

To further investigate the heterojunction of the WSe_2_-graphene, high-resolution transmission electron microscopy (HRTEM) measurement is performed. In Fig. 3a, selected area TEM image indicates that the overlap region between black (graphene end) and green (TMD end) dashed lines is composed of the pre-patterned graphene and the sequential grown WSe_2_ monolayer. Width of the overlapping region is about 500 nm. An amorphous-like TEM image for the graphene lattice is observed as expected because of unavoidable distortions of graphene with the energetic electron beam. Figure 3 c and d present the calculated and experimental observation on the HRTEM image for better understanding on the sequential TMD growth at the heterojunction. Observation of hexagonal lattices and unit cell of graphene (~ 2.5 Å) and WSe_2_ (~ 3.3 Å) is consistent with the parameters in bulk lattices of graphene (2.46 Å) and WSe_2_ (3.28 Å). The TEM characterizations indicate that the sequential WSe_2_ growth initiates at the edges of the pre-patterned graphene because higher defect density at the graphene edge enhances the vertical island growth with more nucleation sites. A large lattice mismatch more than 20% between the lattice of graphene and TMD might be responsible for a disorder interface with higher defect density and for combined vertical and lateral TMD growth at the heterojunction. Moreover, the insets in Fig. 3d show the corresponding diffractograms by fast Fourier transform (FFT) of real space atomic images in the overlap region and graphene region. Only one set of diffraction pattern is observed at the graphene region (left), while two sets of diffraction patterns rotated with a twisting angle of 0.35° are observed at the overlapped region (right). A highly reduced twisting angle between graphene and WSe_2_ lattices implies that the sequential growth of the WSe_2_ favors coherent stacking at the graphene edges.

**Fig. 4:**
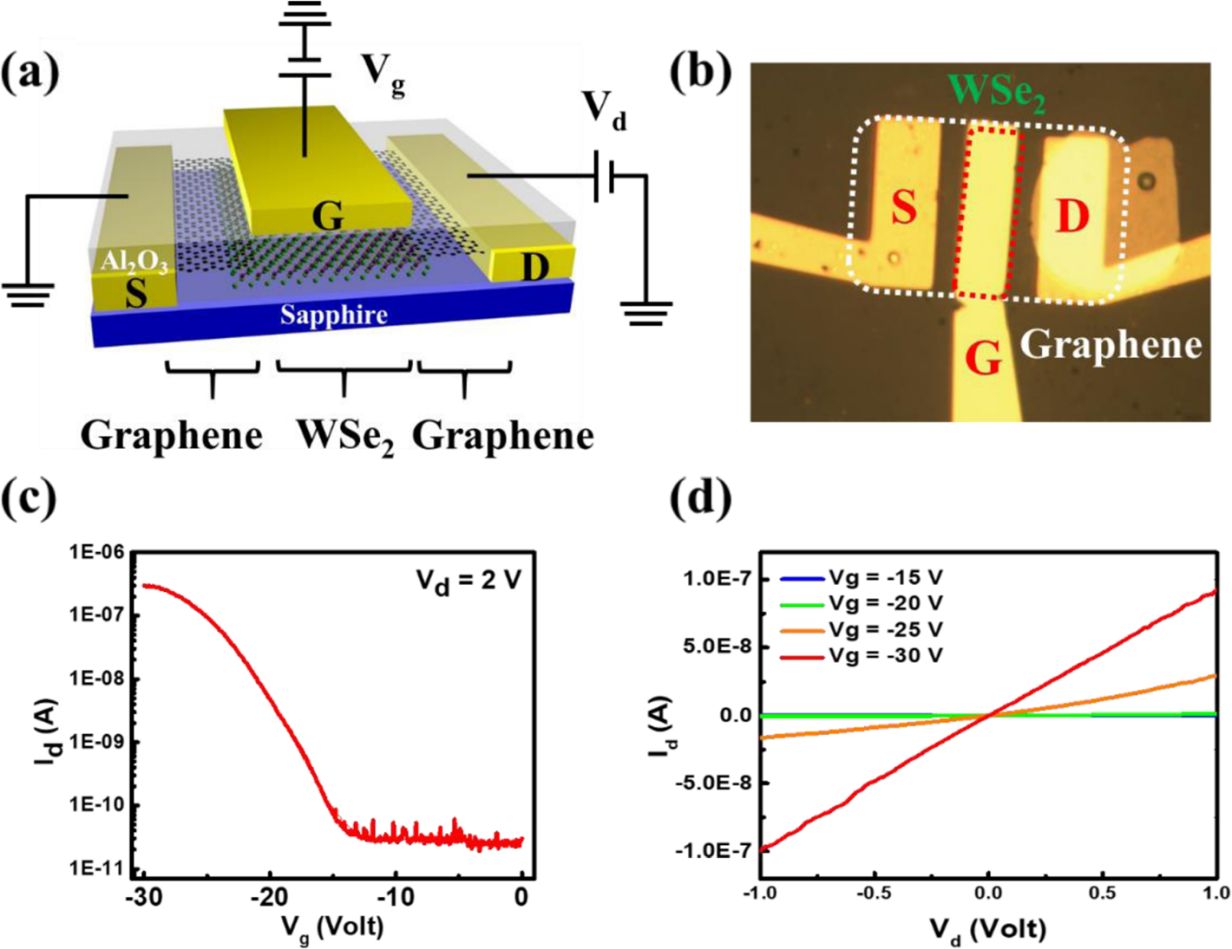
Electronic performance of the WSe_2_ with stitched graphene contacts. **a** The schematic, **b** the optical image, **c** the transfer curve, and **d** output curves of the top-gated monolayer heterojunction device of the stitched graphene-WSe_2_

To demonstrate the field-effect properties of the as-grown WSe_2_ stitched at the edges of patterned graphene hetero-device, the device is fabricated without sample transfer. Customized fabrication process based on surface functionality for e-beam lithography on an insulator is developed. Electronic transport performance of the stitched graphene-WSe_2_ device is studied by connecting metal electrodes (Pd 40 nm) with the patterned graphene and depositing Al_2_O_3_ (50 nm) as gate dielectric. Figure 4 a and b show the schematic illustration of the top-gated heterojunction device and the optical image of the as-fabricated device, respectively. Two-terminal electronic transport measurements are carried out using commercial probe-station (Lake Shore Cryotronics PS-100 with Agilent B1500a) under vacuum at room temperature. The transfer curve of the device exhibits a p-type transport behavior with an on/off ratio (~ 10^4^) and high on-current of approximately a few 100 nA (Fig. 4c). The field-effect mobility of the device in the linear region is around 0.07 cm^2^/Vs at *V*_d_ = 2 V, which is evaluated using the following equation:

1 $$ \mu =\frac{1}{C_{\mathrm{ox}}}\frac{L}{W}\frac{\partial {I}_{\mathrm{D}}}{\partial {V}_{\mathrm{G}}}\frac{1}{V_{\mathrm{D}}} $$

where *C*_ox_ = *ε*_0_*ε*_r_/*d* is the oxide capacitance and *L* (9 μm) and *W* (24 μm) are the channel length and channel width, respectively. Moreover, the output curves of the device at various gate voltages are shown in Fig. 4d. The linear *I*-*V* curves confirm a good contact between graphene layer and WSe_2_ layer. An enhanced electronic performance of the stitched TMD-graphene monolayer heterojunctions is achieved because of improved contact properties, suggesting the synthesis for sequential TMD growth at the edges of artificially patterned graphene moves a significant step towards 2D nanoelectronics.

## Conclusions

Sequential WSe_2_ growth at the edges of the patterned graphene is achieved on sapphire by using promoter-assisted LPCVD. The PTAS promoters significantly reduce growth temperature for ideal sequential WSe_2_ growth at the graphene edges with larger domain sizes.

The TEM characterizations indicate that the sequential WSe_2_ growth initiates at the edges of the pre-patterned graphene. A highly reduced twisting angle between graphene and WSe_2_ lattices implies that the sequential WSe_2_ growth favors coherent stacking at the graphene edges. An enhanced electronic performance of the stitched TMD-graphene monolayer heterojunctions is achieved because of improved contact properties.

## Supplementary information

**Additional file 1: Figure S1.** Scalable synthesis of monolayer WSe_2_ on sapphire: influence on (a) concentration of the seeding promoters and (b) temperature for the WSe_2_ growth. **Figure S2.** Synthesis of the laterally stitched graphene-WS_2_: (a) optical and (b)AFM image of the graphene-WS_2_ and (c) PL spectrum of the WS_2_. Raman mapping of (d) the E_2g_ mode of WS_2_ and (e) the G’ mode of graphene. (f) PL mapping of the Graphene-WS_2_. (Scale bar: 4 μm).

## Data Availability

All data generated or analyzed during this study are included in this published article and its supplementary information files.

## Abbreviations

2D: Two-dimensional
AES: Auger electron spectroscopy
EIS: Electrochemical impedance spectroscopy
SEM: Scanning electron microscopy
TEM: Transmission electron microscopy
TMD: Transition metal dichalcogenides
XPS: X-ray photoelectron spectroscopy
XRD: X-ray diffraction
