# Supplementary material for: Selective Growth of WSe2 with Graphene Contacts
Source: Nanoscale Res Lett. 2020 Mar 12;15:61. doi: 10.1186/s11671-020-3261-y (PMC7067944; doi:10.1186/s11671-020-3261-y)
Supplement: Supplementary file 1 — Additional file 1: Figure S1. Scalable synthesis of monolayer WSe2 on sapphire: influence on (a) concentration of the seeding promoters and (b) temperature for the WSe2 growth. Figure S2. Synthesis of the laterally stitched graphene-WS2: (a) optical and (b)AFM image of the graphene-WS2 and (c) PL spectrum of the WS2. Raman mapping of (d) the E2g mode of WS2 and (e) the G’ mode of graphene. (f) PL mapping of the Graphene-WS2. (Scale bar: 4 μm). [file 11671_2020_3261_MOESM1_ESM.docx]

**Supporting Information for**

**Selective Growth of WSe_2_ with Graphene Contacts**

Yu-Ting Lin,^a^† Xin-Quan Zhang,^b^† Po‐Han Chen,^b^ Erh-Chen Lin,^b^ Jian-Guo Rong,^b^

Yung‐Fu Chen,^*a^ Yi-Hsien Lee^*b^

*^a^Department of Physics, National Central University, Zhongli 32001, Taiwan*

*^b^Department of Materials Science and Engineering, National Tsing Hua University, Hsinchu 30013, Taiwan*

*E-mail: yfuchen@cc.ncu.edu.tw (Yung‐Fu Chen^*a^) yhlee.nthu@gmail.com (Yi-Hsien Lee^*b^)

**
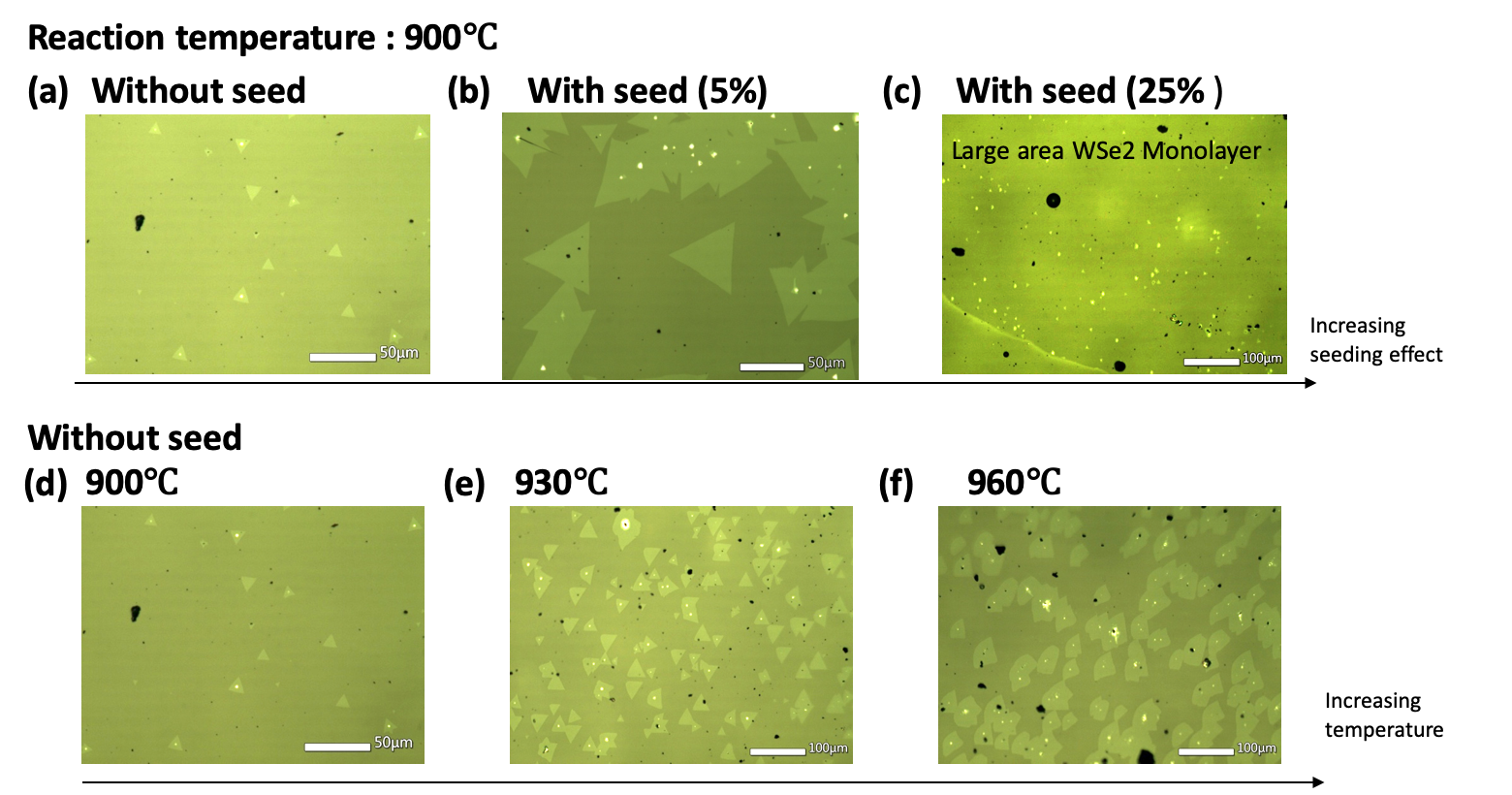
**

Figure S1 Synthesis of large area WSe_2_ monolayers on sapphire: (a-c) the effect of increasing the concentration of seeding promoters. Large area of WSe_2_ monolayer could be synthesized by increasing the nucleation concentration. (d-f) Synthesis of WSe_2_ monolayer without seeding promoters. Substrate-guided growth of irregular WSe_2_ monolayer was observed and increased temperature would result in increased domain size.

**
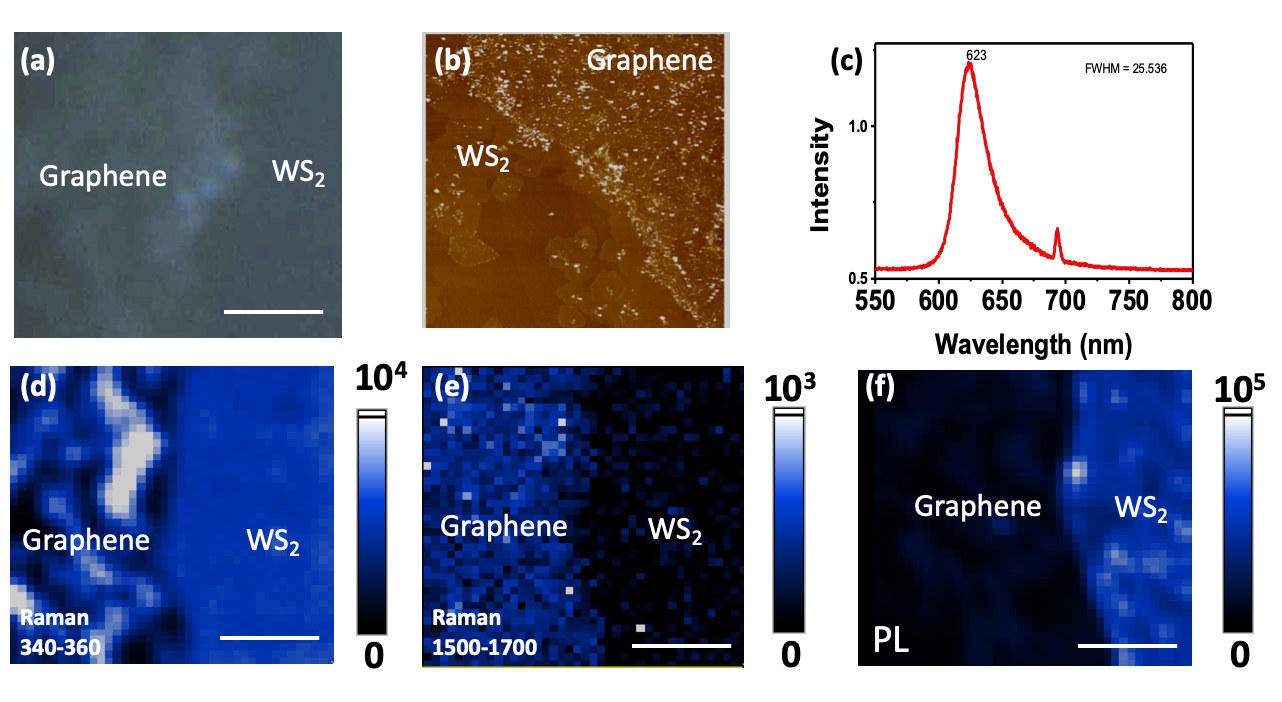
**

Figure S2 Synthesis of monolayer lateral heterostructure of graphene-WS_2_­: (a) optical image (b)AFM image of graphene-WS_2_ lateral heterostructures(c) PL spectrum of WS_2_ monolayer. (d) Raman mapping of E_2g_ mode of WS_2_ (e)Raman mapping of G’ mode of graphene (f) PL mapping of Graphene-WS_2_ lateral junctions. Scale bar: 4µm
